# Polyclonal antibodies for the detection of *Trypanosoma cruzi* circulating antigens

**DOI:** 10.1371/journal.pntd.0006069

**Published:** 2017-11-09

**Authors:** Edith S. Málaga-Machaca, Alessandra Romero-Ramirez, Robert H. Gilman, Sofía Astupiña-Figueroa, Noelia Angulo, Alejandro Florentini, Cinthya J. Lovon-Luque, Remo A. Gonza, Ada del Carpio-Sanz, Inés Cabello, Rosina Camargo, Fernando Recuenco, Liliam A. Barrueta-Soria, Manuela R. Verastegui, Maritza Calderon, Holger Mayta

**Affiliations:** 1 Infectious Diseases Research Laboratory, Departamento de Ciencias Celulares y Moleculares, Facultad de Ciencias y Filosofía, Universidad Peruana Cayetano Heredia, Lima, Perú; 2 Department of International Health, Johns Hopkins Bloomberg School of Public Health, Baltimore, Maryland, United States of America; 3 Asociación Benéfica PRISMA, Lima, Perú; 4 Departamento de Medicina, Facultad de Medicina Humana, Universidad Católica de Santa María, Arequipa, Perú; 5 Departamento Académico de Salud Animal y Salud Pública, Facultad de Medicina Veterinaria, Universidad Nacional Mayor de San Marcos, Lima, Perú; US Food and Drug Administration, UNITED STATES

## Abstract

**Background:**

Detection of *Trypanosoma cruzi* antigens in clinical samples is considered an important diagnostic tool for Chagas disease. The production and use of polyclonal antibodies may contribute to an increase in the sensitivity of immunodiagnosis of Chagas disease.

**Methodology/Principal findings:**

Polyclonal antibodies were raised in alpacas, rabbits, and hens immunized with trypomastigote excreted-secreted antigen, membrane proteins, trypomastigote lysate antigen and recombinant 1F8 to produce polyclonal antibodies. Western blot analysis was performed to determine specificity of the developed antibodies. An antigen capture ELISA of circulating antigens in serum, plasma and urine samples was developed using IgY polyclonal antibodies against *T*. *cruzi* membrane antigens (capture antibody) and IgG from alpaca raised against TESA. A total of 33 serum, 23 plasma and 9 urine samples were analyzed using the developed test. Among serum samples, compared to serology, the antigen capture ELISA tested positive in 55% of samples. All plasma samples from serology positive subjects were positive in the antigen capture ELISA. All urine positive samples had corresponding plasma samples that were also positive when tested by the antigen capture ELISA.

**Conclusions:**

Polyclonal antibodies are useful for detection of circulating antigens in both the plasma and urine of infected individuals. Detection of antigens is direct evidence of the presence of the parasite, and could be a better surrogate of current infection status.

## Author summary

Current diagnosis of Chagas disease is still cumbersome. Diagnosis is based on antibody detection with at least two assays of distinct mechanisms. If a discrepancy exists, a third assay must be performed. However, detection of antibodies is not indicative of current infection. Molecular-based techniques such as qPCR have been used for diagnosis and as a gold standard in the demonstration of therapeutic failure, but availability of genomic material depends on the presence of parasites in the bloodstream. Detection of parasite-derived antigens represents a better alternative for diagnosis, as several proteins are secreted/excreted by the parasites and may be detected in blood and in the urine of infected individuals. This study describes the development of polyclonal antibodies raised against different *Trypanosoma cruzi* antigens and their applicability for the diagnosis of Chagas disease using the widely-used ELISA format.

## Introduction

Chagas disease, caused by the protozoan parasite *Trypanosoma cruzi*, is endemic to many parts of the Americas [1–3]. This parasite infects a wide variety of wild and domestic mammals including humans [2]. The disease is transmitted by insect vectors (members of the Triatominae family) with metacyclic trypomastigotes present in their feces. Parasite trypomastigotes gain access to the tissue and circulatory systems at the bite site, through wounds caused by scratching the bite site, or through the mucous membranes [4]. Other transmission routes include congenital transmission, blood transfusions, organ transplantation, oral transmission through the consumption of food contaminated with feces from infected insects, and accidental laboratory exposure [1, 3, 4].

It has been estimated that Chagas disease affects approximately 8 million people and may cause about 12,000 deaths each year (45,000 in the 1980s and 23,000 in the 1990s) [2,5]. Bolivia is the country with highest endemicity with a prevalence of up to 80–90% in rural areas [6,7].

Two main phases can be distinguished in Chagas disease, the acute and chronic phase, each with different characteristics. The acute phase occurs at the beginning of the infection and is characterized by patent parasitemia with most of the patients asymptomatic. Approximately 75% of acute cases are in children under 10 years old [3,8]. In most cases, patients are asymptomatic (95%), however when the inoculation site is the conjunctiva mucous membrane, the characteristic Romaña’s sign, an eyelid edema, may appear [9,10].

The chronic phase can appear years to decades following the acute phase. This phase is characterized by a very low parasitemia and the most suitable methods for diagnosis are immunological assays [11]. Unlike the acute phase, about one third of infected patients will develop chronic phase symptoms. These symptoms mainly include heart diseases such as cardiomyopathy which is associated with heart insufficiency, sometimes leading to mortality including sudden death in some patients [10, 12]. The disease may also affect the gastrointestinal system causing mega colon or mega esophagus [9, 13].

Diagnosis of Chagas disease is based on clinical and laboratory assessment. Most laboratory assays are dependent on the detection of antibodies. Two positive results, preferably based on serological methods of distinct mechanisms (e.g., whole-parasite lysate and recombinant antigens), are required for an individual to be considered Chagas disease positive [3, 14]. As with most of the of serological assays, these tests are not indicative of current infection when used alone and may cross-react with other parasitic diseases such as leishmaniasis and malaria, depending on the antigen used in the assay [15].

Immunological diagnosis is based on the use of parasite derived antigens. Trypomastigote excreted-secreted antigen (TESA) is used in Western blot (TESA-Blot) for both acute and chronic phase diagnosis, generating a characteristic pattern of bands depending on the strain of *T*. *cruzi* used. TESA-blot is highly specific and sensitive; sera of infected individuals identify protein bands of 130–200 kDa in the acute phase, while sera from individuals in the chronic phase identify protein bands of 150–160 kDa [16]. However, Western blot is not very economical to produce, requiring special training and a sophisticated laboratory.

While antibody detection is indirect evidence of infection, detection of any antigenic fraction of the parasite is considered the equivalent of finding the whole parasite. Antigen detection may even occur prior to development antibodies at detectable levels [17]. Several reports have shown the presence of different proteins of *T*. *cruzi* in the urine of infected animals and humans [18–20]. These proteins have been used to develop monoclonal and polyclonal specific antibodies to be used for antigen detection both in urine and serum samples [17,19,21]. Due to the variety of circulating and excreted antigens, targeting a specific protein decreases sensitivity because the presence of antigens is variable and depends on different factors such as the phase of the disease [21,22], renal injury [20] among others.

The antigen 1F8 is a recombinant protein with a molecular weight of 24–25 kDa derived from a protein found in the flagellum of *T*. *cruzi* [23]. This calcium binding protein is used as antigen in an ELISA for the diagnosis of both acute and chronic Chagas disease [24] with high sensitivity and specificity; but detection of the presence of this protein in sera or urine samples has not been performed.

The use of antigens of the parasite for the production of polyclonal antibodies designated for diagnosis or for therapy has been a very important tool for research. IgY is a type of immunoglobulin, and the major one in birds, with a molecular weight of 180 kDa. This is much larger than the IgG of most mammals, often about 159 kDa [25]. One of the most important characteristics of IgY antibodies is that they are able to recognize different epitopes than the antibodies raised in the mammals usually do [26]. In addition, IgY does not activate the complement system, providing a great advantage when used as capture antibody in immunoassays [27]. Antibodies from camelids (IgG2 and IgG3) because of their lower size probably recognize inaccessible epitopes that may not be recognized by mammalian antibodies [28–30].

To produce polyclonal antibodies, we used three different animals: alpacas, rabbits, and hens and immunized them with different *T*. *cruzi* antigens. The resulting antibodies were then used to develop an antigen capture ELISA and tested for their ability to discriminate and identify individuals infected with *T*. *cruzi* the agent of Chagas disease.

## Materials and methods

### Ethics statement

Human sera, plasma, and urine samples were archived samples obtained from previous studies. The Human Ethics Committee of the Universidad Peruana Cayetano Heredia approved the use of these archived samples.

The Animal Ethics Committee of the Universidad Peruana Cayetano Heredia approved the protocols for the use of animals for antibody production, approval Code 61549—Cons-CIEA-029-2014.

The Animal Ethics Committee of the Universidad Peruana Cayetano Heredia is registered in the Office of Laboratory Animal Welfare, Department of Health and Human Services, National Institutes of Health (NIH—USA) and follows its rules and laws.

The use of animals in this study was performed following the Deontological Code of the Medical Veterinary College of Peru; The Care and Use of Experimental Animals. Canadian Council on Animal Care 1980 and the Australian code of practice for the care and use of animals for scientific purposes 1997.

### Production of *Trypanosoma cruzi* antigens

In order to produce polyclonal antibodies alpacas, rabbits, and hens were immunized using four different antigens: trypomastigote lysate antigen (TLA), trypomastigote membrane proteins (TMP), trypomastigote excretory-secretory antigen (TESA), and a commercial recombinant 1F8 *T*. *cruzi* antigen (Genway Biotech Inc, CA-USA). TLA and TMP were obtained from *T*. *cruzi* Y strain trypomastigotes. Parasites were washed three times using cold PBS at 2,000 x g for 10 min before antigen preparation.

For TLA the parasite pellet was resuspended in 2 ml PBS, frozen and thawed three times using a dry ice-ethanol bath, and sonicated (Misonix, Sonicator 3000) for 4 cycles at 30 s ON, 60 s OFF (Output Power: 3). The suspension was centrifuge at 13,500 x g for 20 min. The supernatant was recovered and used as antigen.

TMP were extracted using a modify protocol [31]. The parasite pellet was resuspended in 200 μl of 10 mM Tris-HCI, pH 7.4, 140 mM NaC1, and 2.0% Triton X-114 (Triton X-114 buffer), the tube was incubated 90 min at 4°C, and centrifuged at 10,000 x g for 15 min at 4°C. The detergent phase, found at bottom of the tube, was mixed with an equal volume of Triton X-114 buffer, incubated 1 min at 37°C, and centrifuged for15 min at 10,000 x g at room temperature. The detergent phase was used as antigen.

The TESA antigen was harvested from *T*. *cruzi* Y strain growth in LLC-MK_2_ cells as previously described [15]. After the sodium dodecyl sulfate-polyacrylamide gel electrophoresis (SDS-PAGE), and transfer to nitrocellulose, the protein band (150–160 kDa) was excised from the nitrocellulose paper using the reaction of chronic positive sera as a reference. The paper was digested by incubating the excised paper with 250 μl of dimethyl sulfoxide (DMSO) per 20 mm^2^ of nitrocellulose for 1h at room temperature on a rocker mixer. After the incubation, 0.05 M carbonate/bicarbonate buffer (pH = 9.6) was added drop by drop until a volume equivalent to the DMSO used for extracting the antigen from the nitrocellulose paper was added. The mixture was then centrifuged at 4°C for 10 min at 10,000 x g and the pellet washed by centrifugation at 4°C for 10 min at 10,000 x g with 0.1 volumes of phosphate buffered saline (PBS, pH = 7.4). The pellet was re-suspended in an equal volume of PBS. After production, all antigens were stored at -80°C (no more than one month) until used.

### Antibody production

Alpacas were initially immunized using complete Freund’s adjuvant and boosted with incomplete Freund’s adjuvant (Sigma—Aldrich, MO-USA). Rabbits and chickens were immunized using one volume of antigen and one volume of Sigma Adjuvant System (SAS, S6322—Sigma—Aldrich). Three, two years old female alpacas (*Huacaya* breed), eight hens (*New Hampshire* breed) and eight, two months old rabbits (*New Zealand* breed) were immunized according to the scheme showed in Table 1.

**Table 1:** Animal immunization scheme.

Animal		Antigen				Immunization Conditions	
		TESA*	TLA	TMP	r1F8	Inoculation conditions	Adjuvant
Alpaca (one per antigen)	First immunization	~5500mm2	400ug	400ug	-	Intradermic, pre-scapular, 6 points of injection	CFA
	3 Booster immunizations	~5500mm^2^	400ug	400ug	-	Intramuscular, pre-scapular, 6 points of injection	IFA
Rabbit (one per antigen)	First immunization	~2750mm^2^	-	100ug	125ug	Intradermic, pectoral muscle	SAS
	3 Booster immunizations	~2750mm^2^	-	100ug	125ug	Intradermic, pectoral muscle	SAS
Hen (two per antigen)	First immunization	~2750mm^2^	-	100ug	125ug	Intramuscular, upper back, 4 points of injection	SAS
	3 Booster immunizations	~2750mm^2^	-	100ug	125ug	Intramuscular, upper back, 4 points of injection	SAS

Animals were immunized with trypomastigote excretory secretory antigen (TESA), trypomastigote lysate antigen (TLA) and trypomastigote membrane proteins (TMP) or recombinant 1F8 antigen (r1F8). Alpacas were immunized with antigens mixed with complete Freund’s adjuvant (CFA) or incomplete Freund’s adjuvant (IFA). Rabbits and hens were immunized with antigens mixed with Sigma Adjuvant System (SAS; S6322-Sigma Aldrich).

*The mm^2^ refers to the area of nitrocellulose excised from the TESA blot.

Alpaca immunization was carried out according to previous references [32–34]. The blood from alpacas and rabbits were collected and centrifuged at 1,100 x g for 10 min to obtain the sera. The sera were stored at -20°C until used. The collected eggs were maintained at 4°C prior to antibody purification.

### Alpaca and rabbit antibody purification

The HiTrap Protein A columns (GE Healthcare Life Sciences, PA, USA) was initially used to separate IgG3 and IgG1 from alpaca. The non-bound fraction was further purified using HiTrap Protein G (GE Healthcare Life Sciences) to separate the IgG2. Rabbit IgG was purified by affinity chromatography using HiTrap Protein A column (GE Healthcare Life Sciences) following manufacturer's instructions.

### Chicken antibody purification

After two months of immunization, all the eggs were pooled according to antigen and time of collection. Egg yolk was separated from the white and proteins were extracted as described before with some modifications [35,36]. Briefly, for one yolk (about 15 ml volume), 30 ml of PBS were added and mixed carefully for 5 min. Then, 15 ml of chloroform were added and mixed again. The mixture was refrigerated at 4°C for 1–2 h then centrifuged at 1,100 x g for 10 min at room temperature. The upper phase was collected and dialyzed overnight with PBS then concentrated using an Amicon YM 100 filter unit (Amicon, Millipore, Darmstadt, Germany). To remove lipid residues and proteins completely, the concentrated samples were further purified with the Pierce Chicken IgY purification kit (Thermo Fisher Scientific) following manufacturer’s instructions. Finally, IgY antibodies were purified using the HiTrap IgY Purification columns (General Electric, Uppsala, Sweden) following manufacturer's instructions.

### SDS-PAGE for the evaluation of purified polyclonal antibodies

To verify the purity of antibodies, SDS-PAGE under non-reducing conditions was performed. Alpaca IgG1, IgG2, and IgG3, and rabbit IgG samples were diluted 1:10 using sample buffer 1 [100mM Tris-HCl (pH = 6.8), 4% (w/v) dodecyl sulfate (SDS), 0.2% (w/v) bromophenol blue, and 20% (v/v) glycerol)]. IgY samples were diluted 1:4 using a sample buffer 2 [62.6mM Tris-HCl (pH = 6.8), 2% (v/v) SDS, 25% (v/v) glycerol)]. After electrophoresis, the gels were stained using 0.25% Coomassie blue.

### Indirect immunofluorescence assay for the evaluation of purified polyclonal antibodies

To corroborate that the antibodies were reacting with the target parts of the parasite an indirect immunofluorescence assay (IFA) was performed using *T*. *cruzi* Y strain epimastigotes. Briefly, epimastigotes were harvested from LIT cultures (liver infusion tryptose medium) and washed by centrifugation at 1,100 x g for 10 min using 1% formalin in PBS and resuspended in PBS to a final concentration of 10^3^ epimastigotes/ml. A total of 20 μl/well of epimastigote suspension was fixed on poly-L-lysine pretreated slides. Fixed epimastigotes were then incubated with each of the purified antibodies at 37°C for 45 min, washed three times with PBS, and incubated with Goat Anti-Llama IgG H&L (FITC), Goat Anti-Rabbit IgG H&L (FITC) or Goat Anti-Chicken IgY H&L (FITC) diluted in PBS, 0.002% Evans blue, and incubated at 37°C for 30 min. Slides were observed at 400X under an immunofluorescence microscope.

### Pretreatment of urine and serum samples for antigen capture ELISA

Three milliliters of urine sample were lyophilized and reconstituted in 300 μl of PBS (pH 7.2).

Serum or plasma samples were pretreated as previously described [37]. Briefly, 50 μl of sample was diluted with 60 μl of PBS, 0.05% Tween 20, 1.0% milk– 0.2% Bovine Serum Albumin (BSA) and heated at 56°C for 30 min.

### Antigen capture ELISA

Initially different combinations of the developed polyclonal antibodies were used to standardize an antigen capture ELISA using TLA spiked urine or sera samples. The final protocol consisted in sensitizing a Nunc Maxisorp 96 well plate (Nunc Nalgene, Rochester, NY), overnight at 4°C, with anti-membrane IgY (4 μg/ml) in carbonate-bicarbonate buffer (pH = 9.6, capture antibody). The plate was washed three times with PBS, 0.05% Tween 20, blocked with PBS, 0.05% Tween 20, 6% semi-skimmed milk, 1% BSA for 2 h at room temperature. Following washing, 100 μl/well of pretreated samples of either urine, serum or plasma samples pretreated were added and the plate was incubated at 37°C for 1 h. The plate was washed again and 100 μl of detection antibody (alpaca anti-TESA IgG) at 4 μg/ml in PBS, 0.05%Tween 20, 1% milk, 0.2% BSA was added and incubated at 37°C for 1 h. After the final wash, goat-anti-llama peroxidase conjugate (Bethyl, Laboratories Inc.) was added at 1: 7500 in PBS, 0.05%Tween 20, 1% milk, 0.2% BSA and incubated at 37°C for 30 min. After washing, the plate was developed using OPD (Sigma FAST Sigma-Aldrich) as substrate for 15 min. The reaction was stopped using 2 M H_2_SO_4_ and the plate was read at 490 nm using the VERSA Max ELISA plate reader (Molecular Devices, LLC, Sunnyvale, CA).

A sample was considered positive if the absorbance (optical density, OD) obtained in the ELISA was higher than the cut-off value. The cut-off value was determined using the mean plus two standard deviations of the absorbance obtained from all samples negative by serology and qPCR including samples from the volunteers.

### Other diagnostic assays

Diagnosis of Chagas disease in human samples was based on serological assays. ELISA was performed using Chagatek Wiener Recombinante v3.0 ELISA (Wiener laboratories, Rosario, Argentina). Western blot analysis was performed using same TESA used for antibody production. Indirect hemagglutination assay (IHA) was performed using the Chagas Polychaco kit (Lemos Laboratories, Buenos Aires, Argentina). Real time PCR (qPCR) was performed using primers and TaqMan probes targeting the nuclear satellite DNA of *T*. *cruzi* as described previously [38–40].

### Clinical samples

Serum (n = 30), plasma (n = 23) and urine samples (n = 6) were archived samples from HIV positive adults. None of the subjects received or was receiving treatment at the moment of enrollment. A sample was considered positive or negative for Chagas disease by serology if they tested positive or negative to all of the following assays: ELISA, TESA blot and IHA, respectively.

All serum samples were collected in Santa Cruz, Bolivia; 18 were positive and 12 negative to Chagas disease by serology.

Plasma samples and urine samples were collected in Cochabamba, Bolivia. Among the plasma samples, 20 were positive and 3 were negative for Chagas disease by serology. Four Chagas-positive and 2 Chagas-negative individuals provided urine samples.

Three serum samples and respective urine samples, obtained from healthy adult volunteers from Lima, Peru (non-endemic for Chagas disease), were included in the analysis. All samples from these volunteers tested negative in all serology assays and with qPCR.

The qPCR was performed using clot samples and phenol chloroform extraction [41]. A sample with a quantification cycle (Cq) equal or greater than 40 was considered qPCR negative.

Cross-reactivity of the Ag-ELISA was determined using serum and plasma samples from adults positive for malaria (n = 5), toxoplasmosis (n = 5) and leishmaniasis (n = 4). Malaria serum samples were positive for *Plasmodium vivax* by both thick blood smear and PCR. Toxoplasmosis plasma samples were from HIV-positive individuals who tested positive for *Toxoplasma gondii* by ELISA (ELISA-IBL international, Hamburg, Germany). Leishmaniasis samples (2 plasma and 2 serum) were from adult subjects infected with mucocutaneous leishmaniasis diagnosed by ELISA and PCR.

## Results

### Antibody production

**Fig 1:**
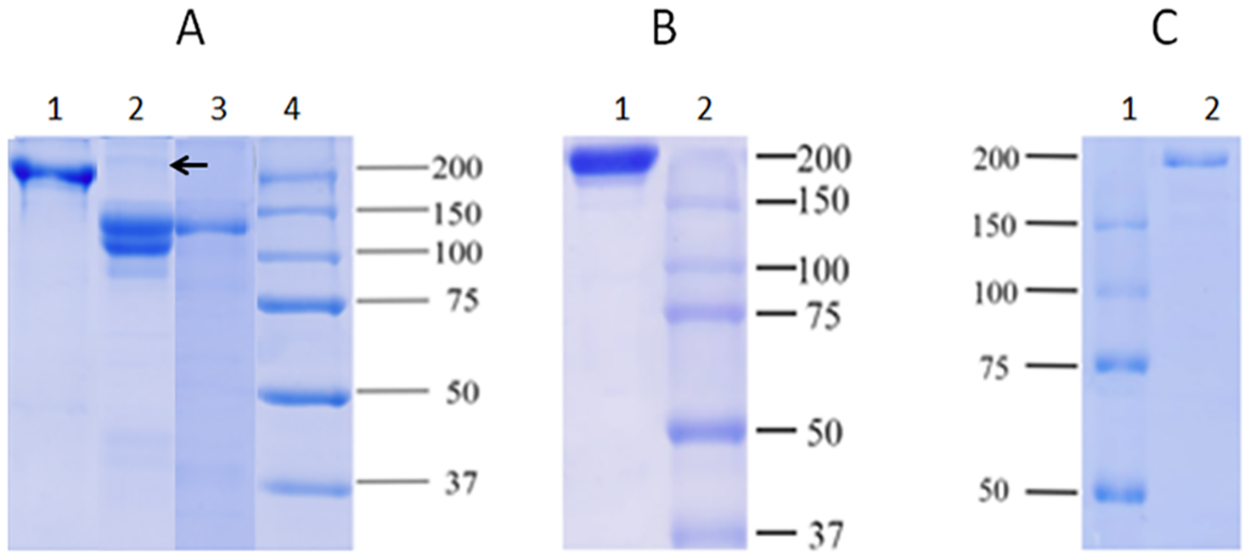
SDS-PAGE of purified antibodies. A: Purified IgGs from alpaca; Lane 1 = IgG1, lane 2 = IgG2, lane 3 = IgG3, lane 4 = molecular weight marker. B: purified IgG from rabbits; lane 1 = IgG, lane 2 = molecular weight marker. C: purified IgY from eggs; Lane 1 = IgY, lane 2 = molecular weight marker. The arrows indicate the presence of IgG1 in the IgG2 purified fraction.

The antibodies purified from alpaca, when analyzed by SDS- PAGE and Coomassie blue stained, showed that the isotypes IgG3 and IgG2 purified have a molecular weight ranging between 100–120 kDa while the IgG1 fraction showed a molecular weight of 170 kDa. The IgG2 fraction was not completely purified, it showed traces of IgG1 (Fig 1A). The IgG purified from rabbits showed a major band at 180 kDa (Fig 1B) while the IgY of chicken showed a major band at 190 kDa (Fig 1C).

**Fig 2:**
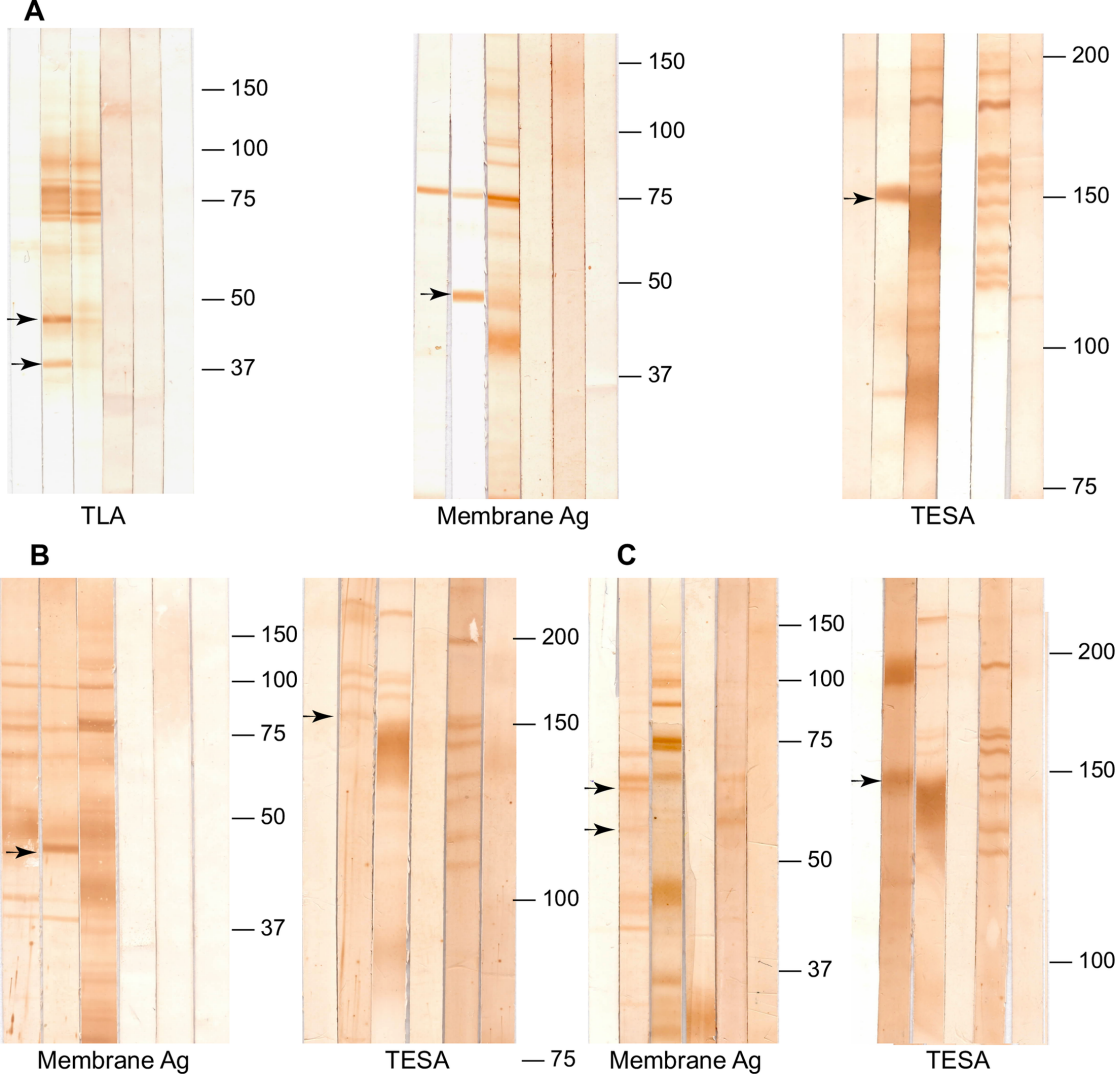
Western blot analysis of polyclonal antibodies. Western blot was performed using 147.7 μg of membrane antigen, 400 μg of trypomastigote excretory secretory antigen (TESA) or 447.7 μg of trypomastigote lysate antigen (TLA). The blot was developed using as primary antibody; A: purified IgG3 from alpaca immunized with *T*. *cruzi* antigens; B: purified IgG from rabbit; and C: purified IgY from eggs. Lane 1 = Pre-Immune IgG/IgY, lane 2 = post-immune purified IgG/IgY, lane 3 = human sera (IgG) from a Chagas positive subject; lane 4 = human sera (IgG) from a Chagas negative subject; lane 5 = human sera (IgM) from a subject with acute Chagas disease; lane 6 = IgM from a human subject negative to Chagas disease. Arrows indicate unique proteins recognized by the developed polyclonal antibodies.

When TLA was used as antigen for western blot and tested with IgG3 from alpaca immunized with TLA, two proteins bands of 40 and 50 kDa were recognized. This pattern of bands was similar to pattern of protein bands recognize by sera from chronic patients. When IgG3 from alpaca was evaluated against membrane antigen, by western blot, it recognized a major band of 50 kDa; the sera of Chagas chronic subjects also recognized this protein band (Fig 2A). The IgG purified from rabbits and IgY purified from eggs immunized with 1F8 antigen detected a band of 25.8 kDa; which did the pre-immune antibodies not recognize (S1A and S1B Fig).

When western blot was performed using TESA, the IgG3 from alpaca, the IgG from rabbit, and the IgY from eggs immunized with TESA recognized a band of 150-160kDa concordant with the SAPA (Shed Acute Phase Antigen) pattern detected by the serum from Chagas acute patients. This protein band also coincides with one of the intense protein bands recognized by the serum of Chagas chronic patients. In addition, the IgG from rabbit and IgY from eggs detected a cross-reacting protein band of 200 kDa also recognized by their pre-immune antibodies (Fig 2B and 2C).

By western blot analysis the evaluation of IgG purified from rabbit and IgY from hens immunized with the membrane antigen, showed similar band patterns of both IgG and IgY pre-immune and post-immune (Fig 2B and 2C). In the IFA, the pre-immune and post-immune IgG from rabbit immunized with the membrane antigen shown similar fluorescent patterns (S2A Fig). In contrast, only post-immune IgY showed fluorescence in the IFA (S2B Fig). Because of these results IgG from rabbits immunized with membrane were not used for further assays.

### Antigen detection in human samples

Among the serum samples the antigen capture ELISA (Ag-ELISA) identified as positive 10 out of the 18 serology positive samples, 9 of these samples were also qPCR positive samples, while all 15 samples negative by both serology and qPCR yield a negative result on the Ag-ELISA (Table 2). Among serum samples, and considering the serology as gold standard, the sensitivity and specificity of the Ag-ELISA were 56% (10/18) and 100% (15/15) respectively.

**Table 2:** Results of the antigen capture ELISA compared to serology and qPCR.

		Serology			
		Pos		Neg	
		qPCR		qPCR	
		Pos	Neg	Pos	Neg
Ag ELISA Serum	Pos	9	1	0	0
	Neg	5	3	0	15
Total serum		14	4	0	15
Ag ELISA Plasma	Pos	16	4	0	0
	Neg	0	0	0	3
Total plasma		16	4	0	3

Samples were considered positive if they tested positive by both TESA Western blot and Chagateck ELISA and may also be IHA positive. Samples were considered positive if their Cq value was equal or less than 39. Only three serum samples were obtained from healthy volunteers, all remaining samples were obtained from HIV positive individuals. Pos = positive, Neg = negative.

None of the samples tested for cross-reactivity tested positive in the Ag-ELISA.

Of the 23 plasma samples only three were negative both by serology and qPCR. The Ag-ELISA was positive for all the serology positive samples (n = 20), four of the serology positive samples were qPCR negative but Ag-ELISA positive (Table 2). Overall, among the plasma samples the sensitivity and specificity of the Ag-ELISA, compared to serology were 100%.

All the urine samples that tested positive to the Ag-ELISA also tested positive on the Ag-ELISA performed in their respective plasma samples (Table 3). Three samples were urine samples collected from volunteers and sera instead of plasma was analyzed; both sera and urine tested negative to the Ag-ELISA.

**Table 3:** Antigen capture ELISA in urine samples.

Sample Code	Serology	qPCR	Ag ELISA OD (plasma)*	Ag ELISA result (Plasma)	Ag ELISA OD (Urine)	Ag ELISA result (urine)
018R	Pos	Pos	0.4516	Pos	0.7164	Pos
074R	Pos	Pos	0.6831	Pos	1.1876	Pos
080R	Pos	Pos	0.5941	Pos	0.5934	Pos
094R	Neg	Neg	0.3638	Neg	0.2033	Neg
098R	Neg	Neg	0.4138	Neg	0.1198	Neg
V001	Neg	Neg	0.3300	Neg	0.4135	Neg
V002	Neg	Neg	0.2381	Neg	0.3506	Neg
V003	Neg	Neg	0.2993	Neg	0.3240	Neg
099R	Pos	Neg	0.6465	Pos	2.0799	Pos

Antigen capture ELISA was performed as describe in the text. The cut off values (mean plus two standard deviations) were 0.3816 for serum samples; 0.4395 for plasma samples; and 0.5195 for urine samples.

*Antigen capture ELISA for samples code V001—V003 were performed using serum samples and were obtained from volunteer individuals. Ag ELISA = antigen capture ELISA, OD = optical density, Pos = positive, Neg = negative.

Antigen detection limit was determined by two-fold dilution of the TLA antigen from 1 μg/ml to 0.975 ng/ml; the detection limit was 3.9 ng/ml.

## Discussion

Chagas disease remains an important health problem worldwide. Diagnosis is based on antibody detection by several methods, however antibody detection is not necessarily indicative of current infection. Thus, antigen detection might be a better determinate of current Chagas infection. To our knowledge, this study represents the first study that demonstrated the usefulness of polyclonal antibodies for the diagnosis of Chagas disease in an ELISA format. We have developed polyclonal antibodies against a variety of *T*. *cruzi* antigens in alpacas, rabbits, and hens (eggs), with adequate sensitivity and specificity as to be employed for antigen detection in clinical samples obtained from infected individuals. A combination of chicken IgY developed against *T*. *cruzi* membrane antigens (capture antibody) with alpaca polyclonal antibodies developed against excretory/secretory antigens (detection antibody) in a sandwich ELISA format was useful for the detection of circulating antigens in sera or plasma samples as well as excreted antigens present in the urine samples of infected individuals.

Development and uses of polyclonal antibodies in rabbits and hens have been widely described before with inherent variations depending on the nature of the antigen and the dose and route of administration [42, 43]. Although only two animals (a pair of hens or rabbits) were used to produce antibodies against each antigen, the immune response of each animal was similar as demonstrated by Western blot analysis. Rabbits did not produce a good antibody response when immunized with *T*. *cruzi* membrane antigens, since the Western blot and IFA analysis showed that the pre-immune response was similar to post-immune response. Probably the lack of immune response to these antigens was inherent to the rabbits and not due to the antigen preparation, since both hens and alpacas produced good antibody response. Moreover, the antibodies produced in hens (eggs) against membrane antigens were used in this study to detect *T*. *cruzi* antigens in clinical samples with high sensitivity and specificity.

The use of heavy chain antibodies (modified into nano-antibodies) has been recently explored against Trypanosomes [44, 45] suggesting that alpacas may be capable of generating an adequate immune response against complex mixtures of *T*. *cruzi* antigens. Here we have shown the usefulness of alpaca antibodies for the detection of circulating antigens in different clinical samples.

Several studies describe the use of antigen detection for the diagnosis of Chagas disease [18, 20, 46–49], but all are oriented to the detection of *T*. *cruzi* antigens in urine samples; and all demonstrate the presence of these antigens by Western blot and complicated pre-analytical handling of urine samples. Although Western blot is a standard technique that is widely used is most developed settings, it is still difficult to access in Chagas endemic settings. ELISA based diagnosis is more accessible and useful in these settings. The methodology presented here is simple, accessible and does not require the use of sophisticated laboratory equipment. Although urine samples were lyophilized in this study for practical reasons, ethanol precipitated of antigens [18] perform similarly. They do not differ in the final result from lyophilized antigens, as tested with TLA spiked urine samples and with the positive control samples. Thus, ethanol precipitation might be a good alternative to lyophilization. Only nine urine samples were analyzed in this study. Although there was a perfect correlation between Ag-ELISA in plasma and urine samples, those results need to be corroborated with a larger number of samples.

Lower levels of antigen were detected via the Ag-ELISA in serum samples as compared to the corresponding plasma samples, even in those samples that were qPCR positive. The reason for this low performance is unclear, although it is probable that the pre-treatment technique (heating of samples) might not liberate the *T*. *cruzi* antigens from the immune complexes associated to this disease [29, 50]. Immune complexes may be trapped within the fibrin clot, lowering the availability of antigen. Alternatively, since the test was performed with archived samples, the antigens may have degraded during the storage process. Further analysis is required to clarify this issue.

A high background was observed on the ELISA technique described here. The cut-off value (mean plus two standard deviations) was the highest for urine samples, and the lowest for serum samples. The high cut-off values may be a consequence of the nature of the antibodies and probably could be improved with the use of better blocking agents and techniques.

We have developed polyclonal antibodies that are useful for the detection of circulating antigens in serum, plasma, and urine samples of human subjects infected with Chagas disease using a simple ELISA technique. The antigen detection strategy described here is a promising methodology for the diagnosis of Chagas disease and, because it detects antigens, may be a good surrogate of current infection. Pretreatment of samples is straightforward, and the ELISA is a simple and more accessible technique than the currently described strategies oriented to the detection of *T*. *cruzi* antigens in urine samples.

## Supporting information

S1 Fig Western blot analysis of polyclonal antibodies against r1F8 antigen. Western blot was performed using 447.7 μg of trypomastigote lysate antigen (TLA). The blot was developed using as primary antibody IgG purified from rabbit immunized with r1F8 antigen (A) or IgY purified from eggs obtained from hens immunized with r1F8 antigen (B). MW = Molecular weight marker; Lane 1 = Pre-Immune IgG/IgY, lane 2 = post-immune purified IgG/IgY.
(TIF)

S2 Fig Immunofluorescence assay of polyclonal antibodies against membrane antigens. *T*. *cruzi* Y strain epimatigotes were identified by indirect immunofluorescence assay using a confocal microscopy. IgG from rabbit (A) or IgY from hens (B) immunized with membrane antigens was used as primary antibody. FITC-conjugated anti-rabbit IgG or hen IgY was used as the secondary antibody respectively.
(TIF)
