# Supplementary figures and images for: Polyclonal antibodies for the detection of Trypanosoma cruzi circulating antigens
Source: PLoS Negl Trop Dis. 2017 Nov 9;11(11):e0006069. doi: 10.1371/journal.pntd.0006069 (PMC5705163; doi:10.1371/journal.pntd.0006069)

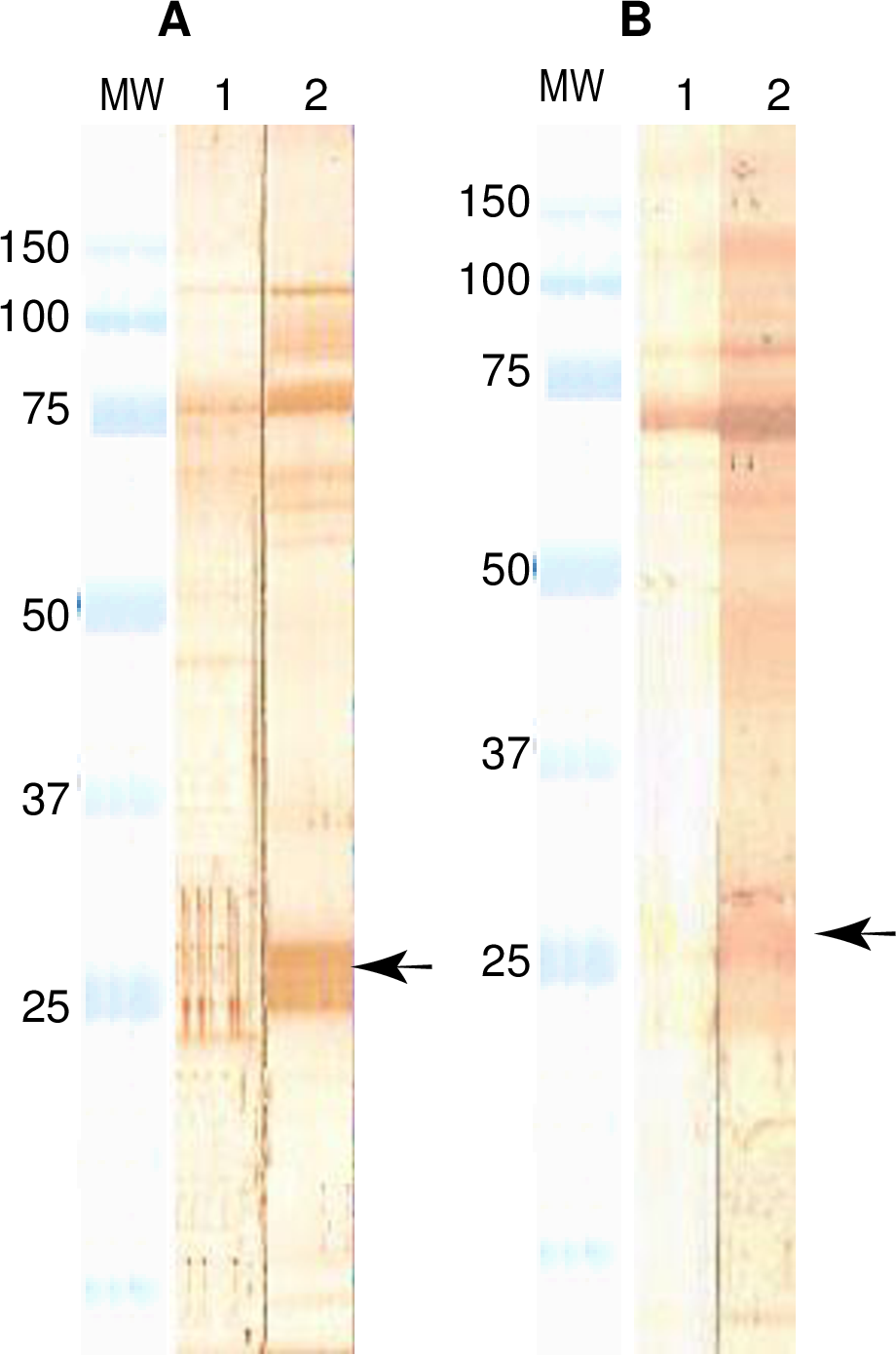

Supplement: S1 Fig — Western blot was performed using 447.7 μg of trypomastigote lysate antigen (TLA). The blot was developed using as primary antibody IgG purified from rabbit immunized with r1F8 antigen (A) or IgY purified from eggs obtained from hens immunized with r1F8 antigen (B). MW = Molecular weight marker; Lane 1 = Pre-Immune IgG/IgY, lane 2 = post-immune purified IgG/IgY. (TIF) [file pntd.0006069.s001.tif]

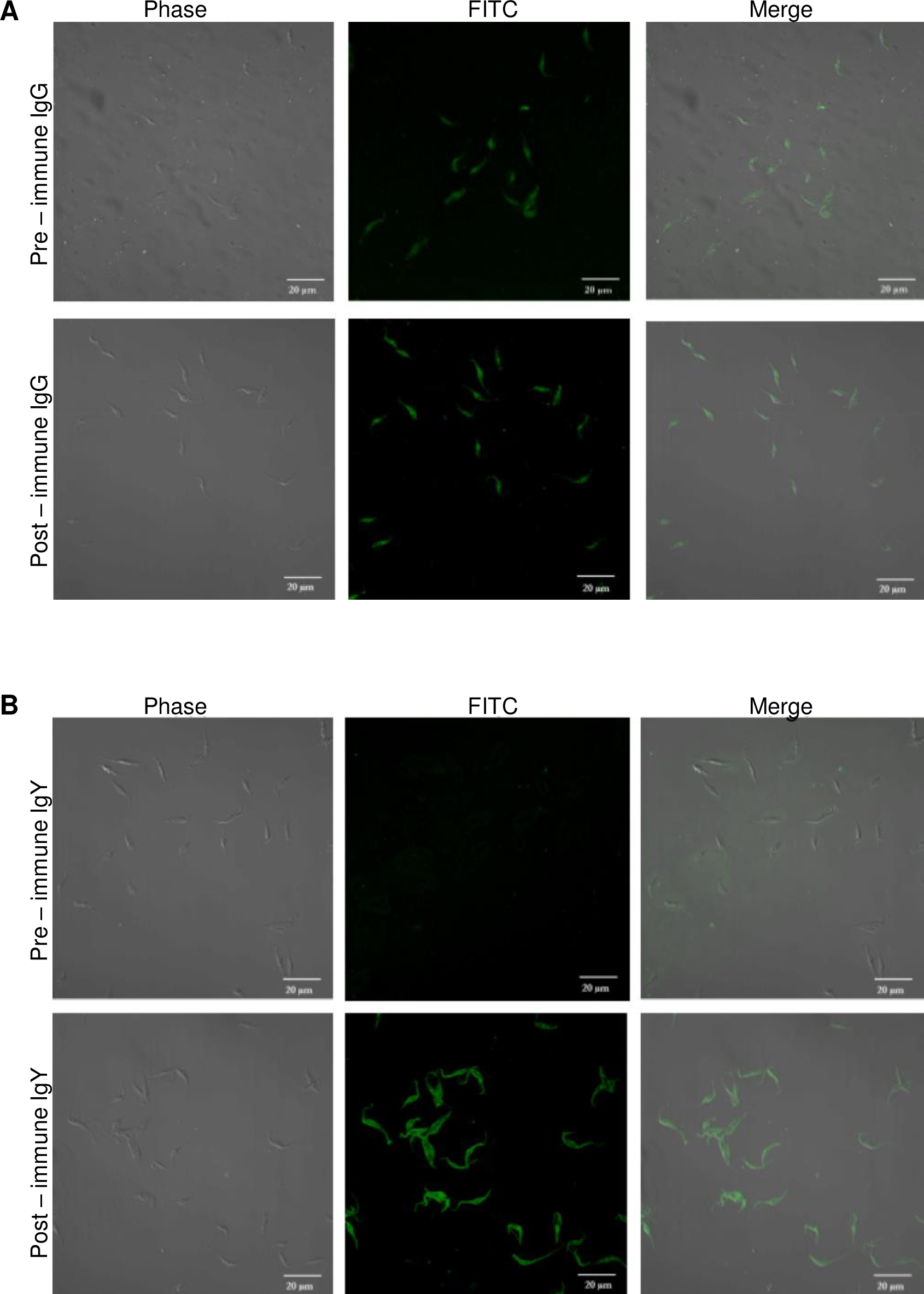

Supplement: S2 Fig — T. cruzi Y strain epimatigotes were identified by indirect immunofluorescence assay using a confocal microscopy. IgG from rabbit (A) or IgY from hens (B) immunized with membrane antigens was used as primary antibody. FITC-conjugated anti-rabbit IgG or hen IgY was used as the secondary antibody respectively. (TIF) [file pntd.0006069.s002.tif]
